# The Identification of Two Head Smut Resistance-Related QTL in Maize by the Joint Approach of Linkage Mapping and Association Analysis

**DOI:** 10.1371/journal.pone.0145549

**Published:** 2015-12-21

**Authors:** Yong-xiang Li, Xun Wu, Jennifer Jaqueth, Dengfeng Zhang, Donghui Cui, Chunhui Li, Guanghui Hu, Huaiyu Dong, Yan-chun Song, Yun-su Shi, Tianyu Wang, Bailin Li, Yu Li

**Affiliations:** 1 Institute of Crop Science, Chinese Academy of Agricultural Sciences, Beijing 100081, China; 2 DuPont Pioneer, Wilmington, DE, 19803, United States of America; 3 DuPont Pioneer, Beijing 101309, China; 4 Maize Research Institute, Heilongjiang Academy of Agricultural Sciences, Harbin 150086, China; 5 Institute of Plant Protection, Liaoning Academy of Agricultural Sciences, Shenyang 110161, China; University of Guelph, CANADA

## Abstract

Head smut, caused by the fungus *Sphacelotheca reiliana* (Kühn) Clint, is a devastating threat to maize production. In this study, QTL mapping of head smut resistance was performed using a recombinant inbred line (RIL) population from a cross between a resistant line “QI319” and a susceptible line “Huangzaosi” (HZS) with a genetic map constructed from genotyping-by-sequencing (GBS) data and composed of 1638 bin markers. Two head smut resistance QTL were identified, located on Chromosome 2 (*q2*.*09HR)* and Chromosome 5 (*q5*.*03HR*), *q2*.*09HR* is co-localized with a previously reported QTL for head smut resistance, and the effect of *q5*.*03HR* has been validated in backcross populations. It was also observed that pyramiding the resistant alleles of both QTL enhanced the level of resistance to head smut. A genome-wide association study (GWAS) using 277 diverse inbred lines was processed to validate the mapped QTL and to identify additional head smut resistance associations. A total of 58 associated SNPs were detected, which were distributed in 31 independent regions. SNPs with significant association to head smut resistance were detected within the *q2*.*09HR* and *q5*.*03HR* regions, confirming the linkage mapping results. It was also observed that both additive and epistastic effects determine the genetic architecture of head smut resistance in maize. As shown in this study, the combined strategy of linkage mapping and association analysis is a powerful approach in QTL dissection for disease resistance in maize.

## Introduction

Among all stresses, maize diseases significantly threaten the achievement of high yield under the inevitable trend of monoculture. Head smut, caused by the fungus *Sphacelotheca reiliana* (Kühn) Clint, constitutes a devastating threat to maize production, especially in regions with low temperatures or at high latitude [1–2]. In Northern China, head smut incidence varied from 7.0 to 35.0% and caused yield loss of up to 0.3 million tons annually [3, 4]. Several factors, including continuous cropping without rotation, planting of susceptible varieties, misuse of seed coating agents and increasing frequency of abnormal weather patterns, have intensified head smut occurrence since 2000. For example, in 2002, the head smut infected area reached about one million hectares, accounting for 19.5% of the total planting area of the Northeast Spring Corn area of China [5]. Head smut is also a severe disease in parts of Southern Europe and North America [6]. Compared with traditional control strategies, such as chemical application and field management practices, the development of disease resistant varieties will be the most convenient, cost-effective and environmentally friendly approach.

Resistance to head smut displays the genetic features of a complex quantitative trait, regulated by small additive genes and their complex interactions [7–9]. Thus, the detection and pyramiding of quantitative trait loci (QTL) for head smut resistance will greatly benefit the efficiency of marker-assisted selection (MAS) in maize breeding. Many head smut resistance QTL had been identified across all 10 chromosomes, including several major and numerous minor loci [2, 10–14]. Two head smut resistant consensus QTL, located in bin 2.09 and bin 3.04, were also identified by Meta-analysis [15]. Moreover, the gene underlying the frequently detected major resistance QTL (*qHSR1*) in bin 2.09 was successfully cloned and is a wall-associated kinase [16].

In addition to QTL identified through linkage mapping, many head smut resistance-related loci have also been detected using the genome-wide association study (GWAS) approach. An association study panel of 80 inbred lines fingerprinted with the MaizeSNP50 BeadChip resulted in 10 SNPs associated with head smut resistance, and the major QTL qHS2.09 was mapped to the interval of less than 1 Mb region when integrating the result of linkage mapping. [14]. In another GWAS study with 144 inbred lines, 18 novel candidate genes associated with head smut resistance were identified in maize [17]. The combination of linkage mapping and association study also appears to be an effective approach in QTL identification for the complex traits, such as disease resistance [14, 18–19].

Chinese inbred lines derived from Mo17, designated as members of the Lancaster group, in general have high levels of head smut resistance and have often been used to map QTL for head smut resistance [12, 14]. Other resistant sources are some Chinese inbred lines selected from the Pioneer commercial hybrid “PH78599”, designated as members of the P group [20], which are also highly resistant to head smut [21]. In this study, inbred line QI319 from the P group, whose high head smut resistance has been demonstrated across multiple environments and years, was used to identify QTL for head smut resistance. Additionally, an association panel with 277 diverse inbred lines [22–23] was used for the association analysis of head smut resistance. The objectives of this study are to: (1) identify QTL conferring head smut resistance using a recombinant inbred line (RIL) population derived from a resistant line “QI319” and a susceptible line “Huangzaosi” (HZS) with the genetic map constructed from genotyping-by-sequencing (GBS) data; (2) validate the head smut resistance QTL in backcross populations; and (3) perform GWAS to detect loci for head smut resistance and to provide evidence for the QTL identified by linkage mapping.

## Materials and Methods

### Plant materials

In our previous study, a RIL population consisting of 143 lines was derived from the cross of the resistant line QI319 and the susceptible line HZS [24]. A series of backcross populations from BC_1_F_1_ to BC_3_F_1_ had been obtained by the continuous backcross of the resistant plants (from 10 to 20 plants in each generation) with the susceptible line HZS under artificial inoculation. To validate the mapping results of the RIL population, a BC_4_F_1_ population with 196 plants was developed by backcrossing eight resistant BC_3_F_1_ plants with the susceptible line HZS. Next, a BC_5_F_1_ population with 1476 plants, obtained from the backcross of 12 resistant BC_4_F_1_ plants containing the target QTL introgression with the susceptible line HZS, was planted and genotyped to find more recombination events. Then, a BC_5_F_2_ population with 138 families, originating from the six founder plants of BC_4_F_1_ population, was planted to further confirm the mapping results. Additionally, an association panel with 277 diverse inbred lines [22–23] was used to validate the head smut QTL by association analysis (S1 File).

### Head smut resistance evaluation under artificial inoculation

The field experiments were separately conducted at the Research Station of Heilongjiang Academy of Agricultural Sciences, Harbin, Heilongjiang Province, China (45.4°N, 126.4°E), the Research Station of Liaoning Academy of Agricultural Sciences, Shenyang, Liaoning Province, China (41.5°N, 123.3°E), and the Changping Research Station of Chinese Academy of Agricultural Science, Changping, Beijing, China (39.5°N, 116.3°E). As agricultural research organizations, all had permission to conduct the activities described in this study. All the experiments were supervised to comply with the local regulations.

The teliospores of *S*. *reliana* were collected from the previous season. Before planting, the teliospores from the sori were mixed with soil at a ratio of 0.1%. For artificial inoculation, the mixture of soil and teliospores was used to cover maize kernels when sowing seeds. The head smut resistance of the RIL population and the BC_4_F_1_ population was phenotyped at the Research Station of Heilongjiang Academy of Agricultural Sciences in 2012. All the RILs were randomly assigned within each of the two replicates with the plot dimensions of 5 m length and 0.6 m apart and were thinned to 25 plants. The BC_4_F_1_ population from the same cross was planted for individual plant head smut resistant phenotyping and genotyping. The resistant BC_4_F_1_ plants were backcrossed to obtain BC_5_F_1_ generation. Using these RILs, two major head smut resistance-related QTL were mapped to Chromosome 2 and Chromosome5, and the major QTL on Chromosome 2 exactly overlapped with the reported locus *qHSR1* [12–14] which was previously map-based cloned [16]. Thus, we focus our work on resolving the other resistance-related QTL located on Chromosome 5. According to the genotypes of BC_4_F_1_ individuals, seeds of the BC_5_F_1_ carrying the target resistance QTL region on Chromosome 5 were planted and self-pollinated to obtain BC_5_F_2_ under the disease-free environment at Changping Research Station of Chinese Academy of Agricultural Science in 2013. Each plant of the BC_5_F_1_ was genotyped for the selection of BC_5_F_2_ with or without the introgression of target QTL. The head smut resistances of 138 BC_5_F_2_ families were scored at the Research Station of Liaoning Academy of Agricultural Sciences in 2014. Each plant of the 38 BC_5_F_2_ families with the heterozygous introgression in the target QTL region was also genotyped and its disease resistance recorded. Finally, head smut resistance of the association panel was recorded in two environments, Shenyang and Harbin in 2009, where two replicates of 277 inbred lines were planted with the plot dimensions of 5 m length and 0.6 m apart. The head smut resistance was weighted by the percentage of infected plants within each plot, defined as disease incidence.

### Genotyping of RILs, BCs and the association panel

Genomic DNA was extracted from leaf tissues using the CTAB method [25]. The RIL population was genotyped using genotyping-by-sequencing (GBS) technology [26]. Each plant of the BC_4_F_1_ population was genotyped using a DuPont Pioneer set of 191 SNPs that uniformly covers the genome [24]. Marker positions were projected on the IBM2 2008 neighbors reference map (http://www.maizegdb.org/). BC_5_F_1_ and BC_5_F_2_ plants were genotyped using another set of 18 SNPs around the resistance QTL region. The association panel was genotyped with the MaizeSNP50 BeadChip [23]. A total of 55,126 SNPs were called successfully among the association panel. SNPs with missing rate of more than 20% and minor allele number (MAF) of <0.05, as well as those with ambiguous physical position, were excluded from the genotyping dataset. Finally, the integrated genotyping dataset that included 41,819 SNPs was obtained.

### Genetic map construction and QTL mapping

For the RIL population, single nucleotide polymorphism (SNP) sites from GBS with minor allele frequency (MAF) <0.05 were first filtered out. Then, the draft parental genotypes were inferred from the low coverage SNP datasets of the RIL population using a maximum parsimonious inference of recombination (MPR) method, and the genotype assignment of each RIL was performed using a hidden Markov model (HMM) approach [27]. For each RIL, consecutive SNP sites with the same genotype were lumped into blocks, a breakpoint was assumed at the transition between two blocks, and markers co-segregating within a block were combined into a recombination bin [28]. The genetic map of the RIL population was constructed from bins serving as genetic markers using the R/qtl package function est.map with Haldane map method [29]. A total of 1638 bins were identified based on the GBS data. Using bin genotypes, the map of the RIL population covers all 10 maize chromosomes with a total genetic distance of 1729.1 cM, and the average bin interval is 1.1 cM. The detailed genetic map used in this study is described in Li et al. (2015) [30].

QTL analysis for the RIL population and the BC_4_F_1_ was conducted with the method of inclusive composite interval mapping (ICIM) in QTL IciMapping software Version 3.3 [31]. For the BC_4_F_1_ population, there were only two genotypes for individuals (HZS/HZS and HZS/QI319), which was similar to the RIL population, and the same method was used for QTL mapping with the BC_4_F_1_ population. The LOD threshold of 2.5 was obtained by 1,000 permutations at a significance level of *P* = 0.05. The correlation of the phenotypic values with different genotypes of the BC_5_F_2_ single plants and the BC_5_F_2_ families was determined by the module of PROC GLM in SAS [32].

### Association mapping

The best linear unbiased predictions (BLUPs) of head smut disease incidence for the association panel across two environments were calculated with the MIXED procedure in SAS (SAS Institute Inc.), within which genotype, environment and replication were treated as random variables [33].

For GWAS with 41,819 SNPs, four models, including the general linear model (GLM) with or without PCA and the mixed linear models (MLM) of both K and PCA+K model, were selected to correct for false positives. Both the GLM and MLM models were processed in TASSEL V4.2.1 [34]. Quantile-quantile plots were shown with a negative log *P*-value of the observed *P*-value from the genotype-phenotype association and the expected *P*-value. The Bonferroni test (0.05/numbers of tests) criterion would be a strict threshold when a large number of markers were used in GWAS [17]. Thus, a lower threshold of–log_10_ (*P*-value) = 5.5 was used as a threshold (*P*-value < 3.61×10^−6^). The epistatic interaction between each pair of resistance-associated SNPs was tested by fitting each marker pair to a general linear model as y = m_*i*_ + m_*j*_ + m_i_*m_*j*_ + error, where y is the head smut disease incidence, m_*i*_ is the effect of the *i*th marker within the tested pair, m_*j*_ is the effect of *j*th marker, m_i_*m_*j*_ is the epistatic effect between the *i*th and *j*th marker, and the error is the residual. If there was significant contribution for m_i_*m_*j*_ in the model (*P*<0.05), a significant epistatic marker pair was supposed to be tested. Linkage disequilibrium (LD) within the QTL region was evaluated using the software of Haploview [35].

## Results

### QTL mapping of head smut resistance with the genetic map constructed from GBS data

**Fig 1:**
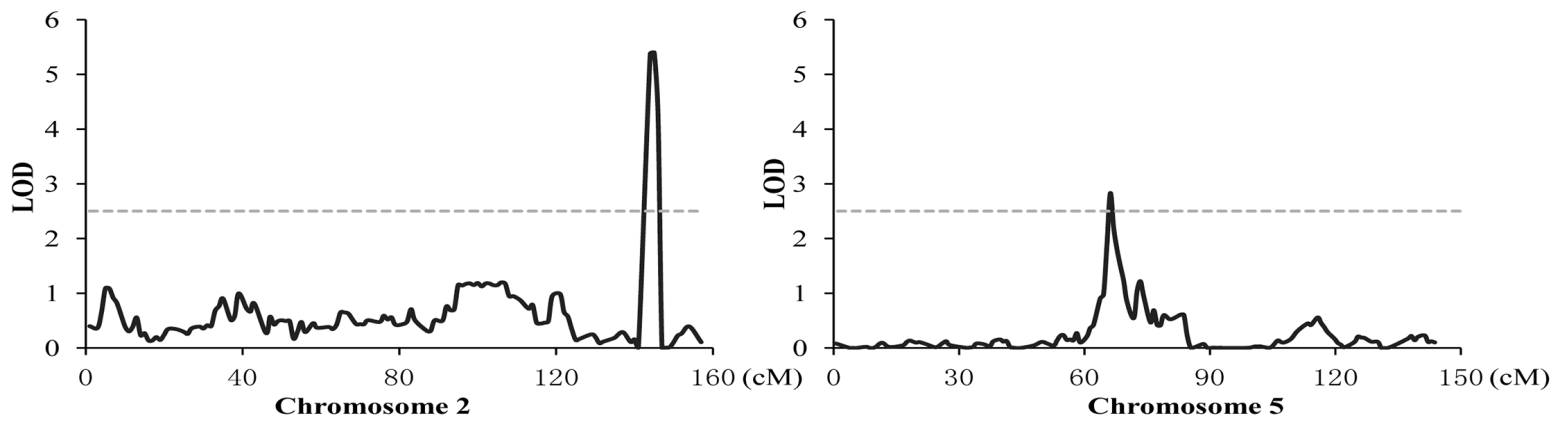
Distributions of head smut resistance QTL on chromosome 2 and 5 for the RIL population of QI319×HZS.

Using the genetic map with 1638 bin markers, two head smut resistance-related QTL, located on Chromosome 2 and 5 and designated as *q2*.*09HR* and *q5*.*03HR*, were identified in a Qi319 x HZS RIL population. The resistant alleles of both QTL are contributed by the resistant parent QI319 (Table 1, Fig 1). Using the genetic map constructed from bin markers, the resistance QTL *q2*.*09HR*, explaining 19.9% of the disease incidence variation for the RIL population, was mapped to the position between the two bin markers of M2_231413244- M2_232886240, which covers about 1.4 Mb on B73 RefGen_v2 sequence (231.4–232.8 Mb) and highly overlaps with a previous fine mapped QTL with the physical region of 231.4–232.4 Mb [14]. Therefore, we assumed that *q2*.*09HR* was the same QTL *qHS2*.*09* that had been successfully cloned by the map-based approach [16]. It should be noted that the RIL population was only evaluated under one environment; however, the results were highly consistent with previous reports. Another head smut resistance QTL *q5*.*03HR* was mapped to the marker interval of 70.8–80.8 Mb on Chromosome 5, which is near the centromere. *q5*.*03HR* appears to be a minor QTL, explaining 4.8% of the phenotypic variance.

**Table 1:** QTL mapping of head smut resistance using the RIL population from the cross of QI319 and HZS.

QTL	Chromosome	Marker interval	LOD value	PVE (%)	Add
***q2*.*09HR***	2	M2_231413244—M2_232886240	5.4	19.9	-12.3
***q5*.*03HR***	5	M5_070870568—M5_080835451	2.8	4.8	-5.1

Add: additive effect of the QTL; negative value indicate that the allele for increasing head smut resistance is contributed by resistant parent QI319.

### Validation of head smut resistance QTL in backcross populations

To validate the effects of the detected QTL, a BC_4_F_1_ population was developed by continuous backcrossing of the head smut resistant plants to the susceptible recurrent parent HZS. Subsequently, the genotypes and phenotypes of 196 BC_4_F_1_ plants were obtained. According to the genotypes of BC_4_F_1_ population, the genetic introgression across the genome was first estimated by the portion of heterozygote in all 191 sites (calculated as the heterozygote number of all sites in 196 plants divided by the total number of all sites in 196 plants). 8.1% of the genetic regions of this BC_4_F_1_ population were revealed as the heterozygous genotype. Different heterozygote portions were observed among different chromosomes, ranging from 3.8% in Chromosome 10 to 14.2% in Chromosome 5. The top two linkage groups for heterozygote portion were observed on Chromosome 2 (10.3%) and 5 (14.2%). Within the regions of the two mapped QTL, the heterozygote portions were 18.8% for *q2*.*09HR* and 18.4% for *q5*.*03HR*, which was much higher than the averaged value across the genome.

**Fig 2:**
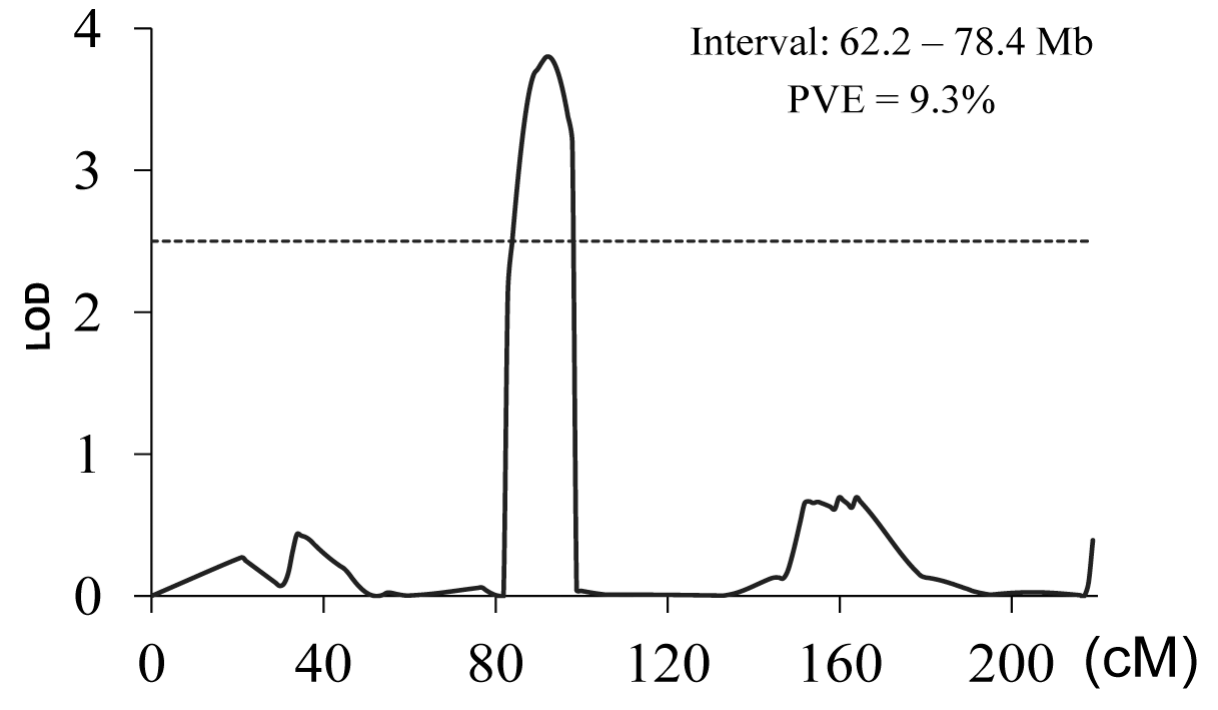
Validation of head smut resistance QTL *q5*.*03HR* with a BC_4_F_1_ population.

**Fig 3:**
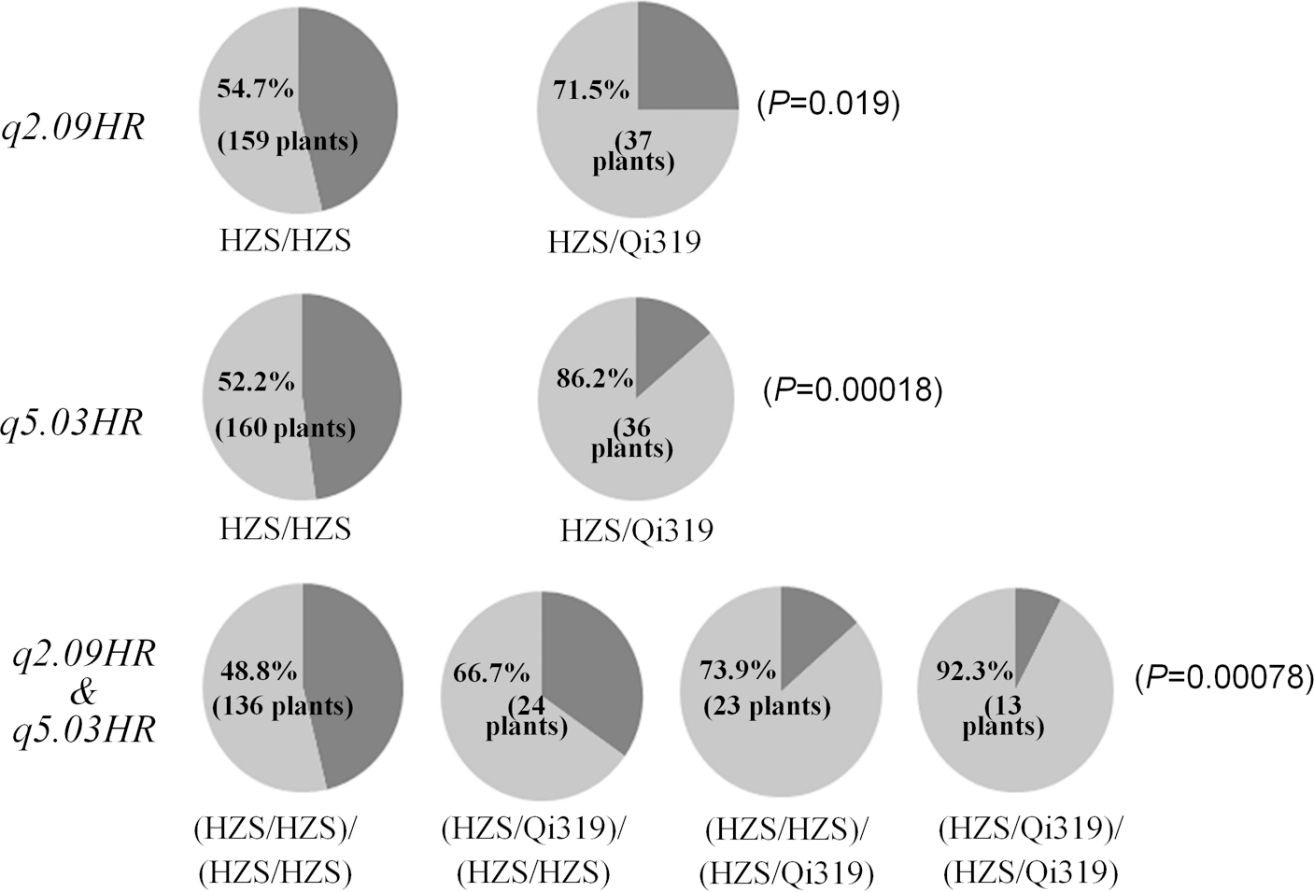
Genetic effects of head smut resistant QTL *q2*.*09HR* and *q5*.*03HR* in BC_4_F_1_ population. HZS stands for the allele contributed by susceptible parent HZS, QI319 stands for the allele contributed by the resistant parent QI319, the lighter gray part in the pie stands for the percentage of resistant plants within each group with the same genotype, the bracketed numbers stands for the total plants of each group. The probabilities of Analysis of Variance (ANOVA) among different allele combinations are noted in the brackets of pie right.

Using the software of QTL IciMapping, a head smut resistance QTL was mapped to the region near the centromere on Chromosome 5 (62.2–78.4 Mb), which could explain 9.3% of phenotypic variation of BC_4_F_1_ population (Fig 2). Thus, combining the mapping results of the RIL and BC populations, both the position and effect of *q5*.*03HR* have been confirmed, which was located within the marker interval of 70.8–78.4 Mb on Chromosome 5. The genetic effects of both *q2*.*09HR* and *q5*.*03HR* were estimated in the BC_4_F_1_ population (Fig 3). Either *q2*.*09HR* or *q5*.*03HR* alone could significantly increase the percentage of resistant plants. A total of 13 BC_4_F_1_ plants were identified to carry the resistant alleles at both loci, and only one individual was identified to be infected by *S*. *reliana*, implying an additive effect of the two QTL.

**Fig 4:**
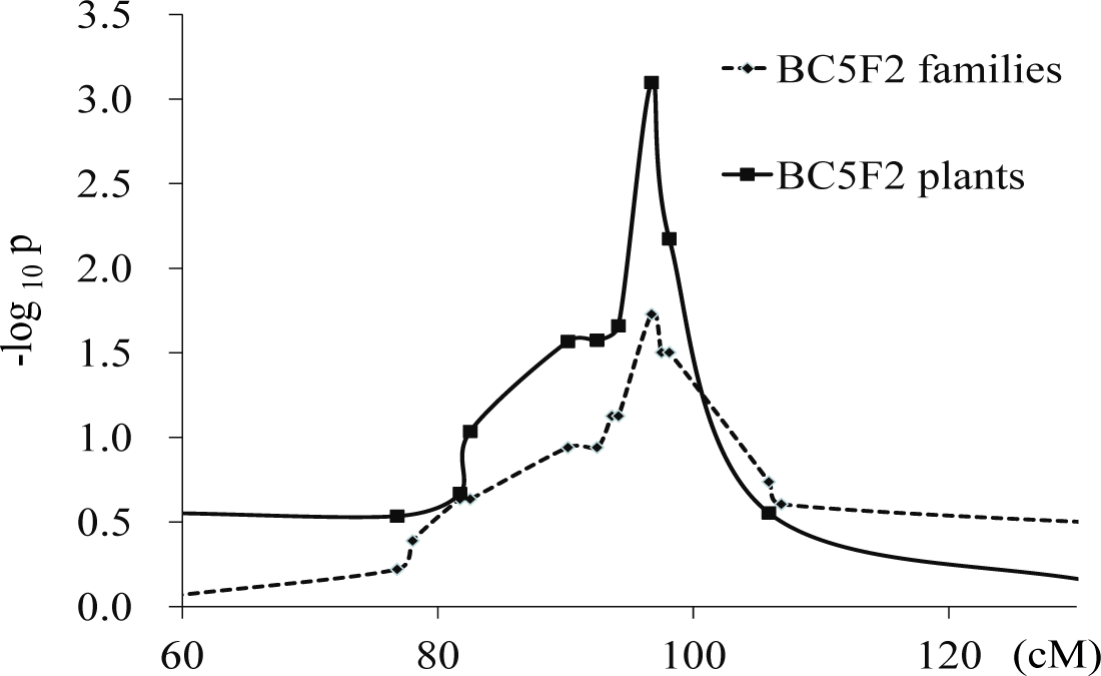
Single marker-trait testing for both the BC_5_F_2_ families and plants. The x-axes represents the genetic position of tested SNPs within the *q5*.*03HR* region, and the y-axes represents the negative log P value of t-test.

To further validate the effect of *q5*.*03HR*, 138 BC_5_F_2_ families with (68 families) or without (70 families) the targeted introgression region were selected and evaluated, among which 603 BC_5_F_2_ individual plants from 38 families with the *q5*.*03HR* introgression were also genotyped and scored for head smut infection. By the single marker-trait testing, significant association could be detected in the single plant panel, as well as the weak association for the BC_5_F_2_ family panel (Fig 4). M5798-39, a SNP marker located at the physical position of 71.8 Mb, showed the most significant association and could explain 3.6% and 3.9% of phenotypic variation in the BC_5_F_2_ family panel and the single plant panel, respectively. The percentages of infected plants within the three groups with the genotypes of HZS/HZS, HZS/QI319 and QI319/QI319 at *q5*.*03HR* for the 603 BC_5_F_2_ plants were 55.6%, 49.4% and 34.7%, respectively, which performed as a dominant or partial dominant locus. Thus, the effect of QTL *q5*.*03HR* could consistently be detected across different environments and generations, although it may only play a minor role under certain conditions.

### The dissection of architecture of head smut resistance by GWAS, as well as the validation of the mapped QTL

Head smut resistance of the association panel was recorded across two environments with two replicates. The BLUPs of head smut disease incidence for the 277 inbred lines were estimated by a mixed model (S1 File). According to the method of Hallauer and Miranda [36], the broad-sense heritability (*h* ^*2*^) of head smut disease incidence for the association panel is 88.7%, which reflects the strong genetic control of head smut disease resistance (Table 2).

**Table 2:** Estimates of components of variance and heritability for head smut infected percentage of association panel with 277 inbred lines after artificial inoculation with *Sphacelotheca reiliana* in two environments.

Parameter	Infected percentage
Variance components	
Genotypes (G)	0.71**
Environments (E)	<0.01
Replications (R)	<0.01
G × E	0.11**
G × R	0.06
Broad-sense heritability (%)	88.7

** Significant at *P*<0.0001.

**Fig 5:**
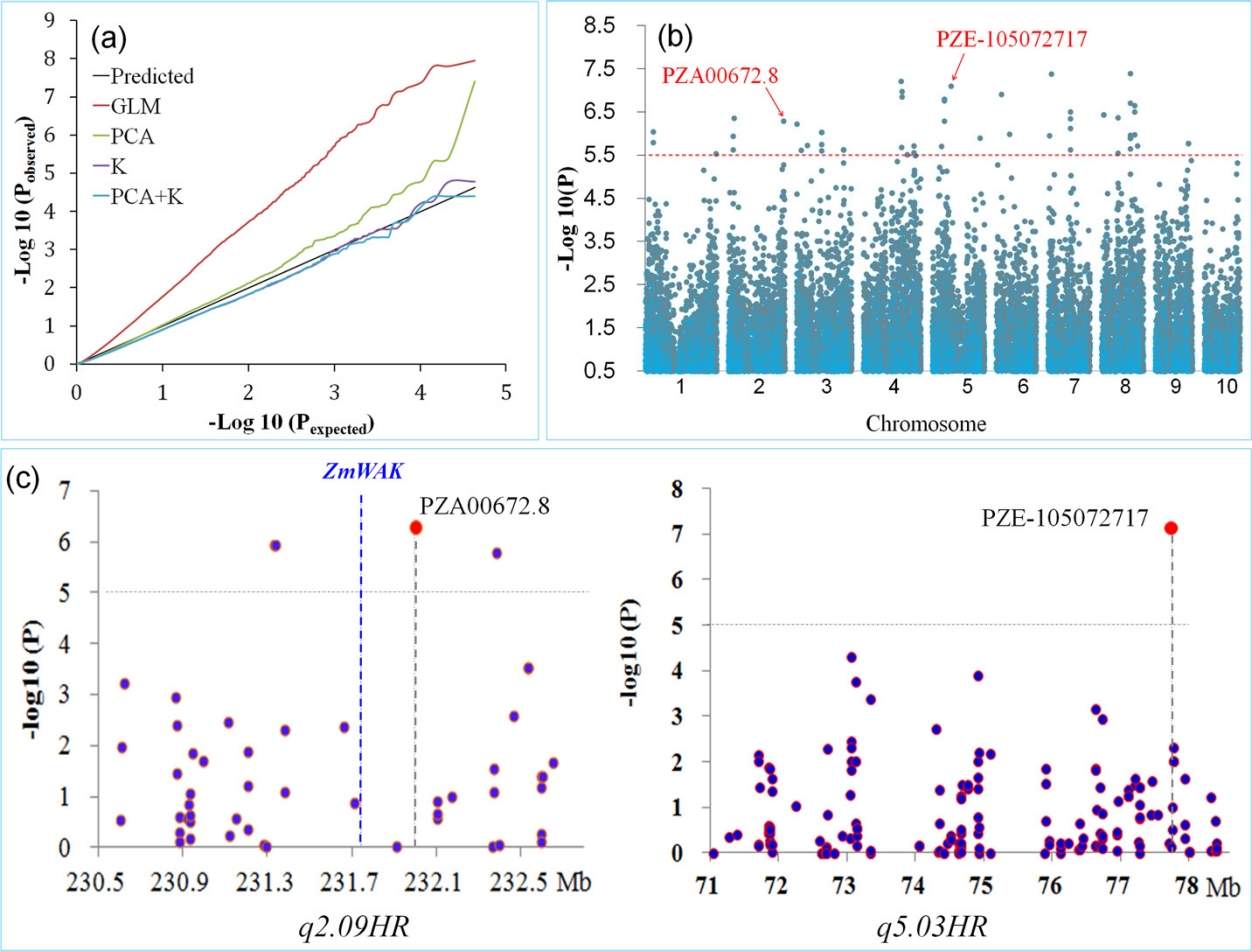
Detection of marker-resistance associations within the regions of *q2*.*09HR* (a) and *q5*.*03H* (b). The horizontal dashed line shows the significance with stringent threshold of–log_10_ (0.00001).

Both the GLM and the MLM were used to perform the association analysis. The results revealed that few significantly associated SNPs were detected from the MLM (including both K and PCA+K models) (Fig 5A). Therefore, the GLM with the control of the first three PCAs was selected for the association study, by which a total of 58 associated SNPs were detected (S2 File, Fig 5B). When the clustered SNPs were considered (within 1 Mb window), a total of 31 head smut resistance-related regions were identified, including the regions of two mapped QTL in this study. A total of 43.1% of the total phenotypic variation was explained when all the most significantly SNPs for each resistance-related region were fit to the general linear model, which was a much lower value than the obtained broad-sense heritability. Therefore, the interactions between each pair of the most significantly associated SNPs within the 31 regions were also tested, and 58 SNP pairs were detected to significantly interact for resistance to head smut (S3 File). When all the interacted pairs were included, the full model of associated SNPs explained 64.1% of the total phenotypic variation, which obtained a much higher value than that of the simple additive model and also revealed that epistasis played important role in the head smut resistance.

**Fig 6:**
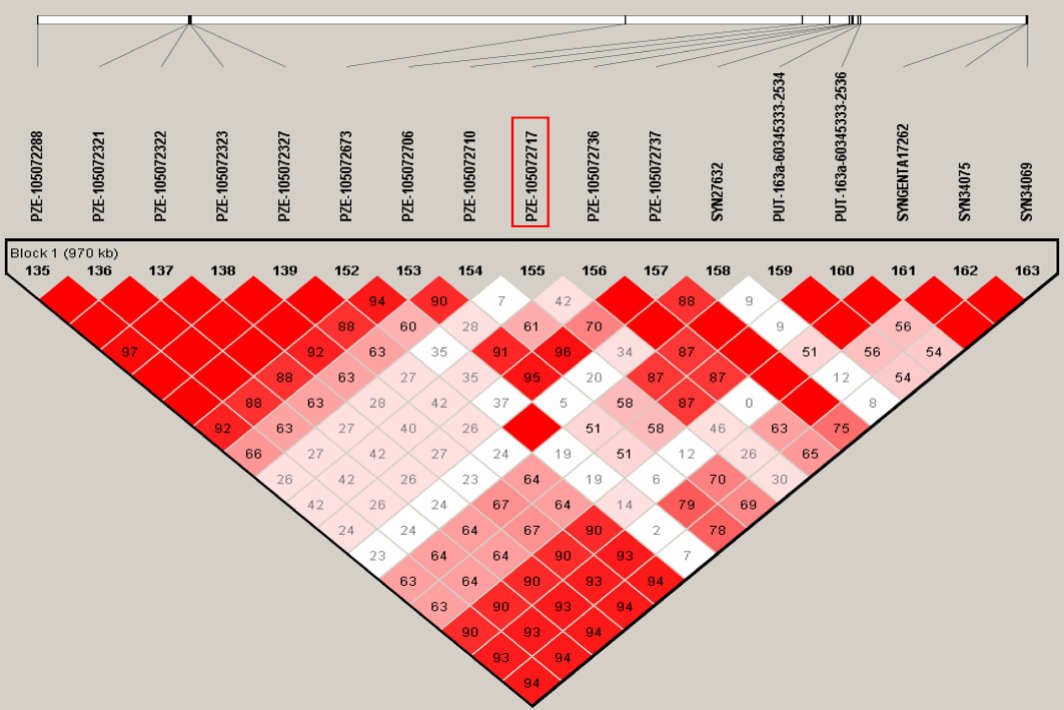
LD heat-map for the region around the most significant SNP PZE-105072717 in *q5*.*03HR*. Numbers for each entry are the linkage disequilibrium (LD) level evaluated with D’. Colors varied from white to deep red mean that the LD level in pair-SNPs is increasing from 0% to 100%. The markers within triangle cycles are tightly linked together (with D’ more than 90%).

Forty-five SNP markers within the *q2*.*09HR* marker interval and 175 SNP markers within the *q5*.*03HR* marker interval were involved in the GWAS, respectively. Four and one SNPs significantly associated with head smut resistance were detected within *q2*.*09HR* and *q5*.*03HR*, respectively (Table 3, Fig 5C). For the four associated markers located at an 1 Mb interval (231.3–232.3 Mb) on Chromosome 2, the peak SNP PZA00672.8, 329 kb downstream of *ZmWAK* [16], has the strongest association with head smut resistance (*P* = 5.32E-07) and explains 9.9% of disease variance in the association panel. Within the region of *q5*.*03HR*, the SNP marker PZE-105072717 shows strong association with disease resistance (*P* = 7.40E-08, PVE = 9.9%) (Table 3, Fig 5C). LD analysis within *q5*.*03HR* revealed that the most significant SNP (PZE-105072717) was located within a 970 kb LD block (Fig 6). The composite effect for the most associated SNPs from *q2*.*09HR* and *q5*.*03HR* was estimated. The two most significantly associated SNPs for each QTL, PZA00672.8 and PZE-105072717, could explain 15.2% of the total disease variation for the association panel, when fit to a full general linear model with the alleles of two loci as classified variables. However, no significant interaction could be detected between them. The genetic effects of both PZA00672.8 and PZE-105072717 were also estimated (Table 4). Inbred lines with the QI319 alleles at both loci have an averaged disease incidence of 37.8%, which is significantly lower than that of lines with the susceptible parent HZS alleles (63.6%) at the alpha level of 0.05. Meanwhile, inbred lines carrying one of the two resistant alleles show intermediate head smut resistance, with the disease incidence of 50.7% for PZA00672.8 and 54.1% for PZE-105072717.

**Table 3:** Significant SNPs associated with head smut resistance within the regions of *q2*.*09HR* and *q5*.*03HR*.

Marker	Alleles	Chromosome	Physical position(Ref. v2)	Marker_*P*	Marker_*R* ^*2*^
**PZE-102187385**	C/A	2	231,332,889	1.17E-06	0.082
**PZE-102187403**	G/A	2	231,338,761	1.17E-06	0.082
**PZA00672.8**	G/A	2	232,004,713	5.32E-07	0.099
**PZE-102188421**	A/G	2	232,381,937	1.59E-06	0.080
**PZE-105072717**	A/G	5	77,746,333	7.40E-08	0.099

**Table 4:** Multiple Comparison analysis on head smut disease incidences among different allele combinations for the most significant SNPs within the regions of both *q2*.*09HR* (PZA00672.8) and *q5*.*03HR* (PZE-105072717) in the association panel.

Allele combination	PZA00672.8	PZE-105072717	Number of lines	Duncan Grouping*	Mean
**QI319/QI319**	GG	AA	190	A	37.8%
**QI319/HZS**	GG	GG	46	B	50.7%
**HZS/QI319**	AA	AA	16	AB	54.1%
**HZS/HZS**	AA	GG	25	C	63.6%
**QI319/-**	GG	-	236	A	40.4%
	AA	-	41	AB	59.9%
**-/HZS**	-	AA	206	A	39.1%
	-	GG	71	AB	55.2%

“*” meant statistical significance at Alpha level of 0.05.

Among the 277 inbred lines in the association panel, 16 of them were directly selected from PH78599 and identified as inbred lines of the typical P group, which includes the resistant parent Qi319. Meanwhile, 19 lines were identified as inbred lines of the TSPT group, which includes the susceptible parent HZS (Table 5) [23]. All the 16 inbred lines from the P group possess the same allele combination at the loci of PZA00672.8 and PZE-105072717 (GG/AA) and showed an averaged disease incidence of 30.5%. Among those 19 TSPT inbred lines, nine possess the allele combination of the susceptible parent HZS (AA/GG). In addition, there were three TSPT inbred lines with the allele combination of the resistant parent (GG/AA), which have much lower head smut disease incidence (32.2%) than those lines with allele combination of the susceptible parent (62.5%).

**Table 5:** BLUPs of infected percentage for the typical lines of two sub-groups within the 277 lines panel that related with QI319 (P) and HZS (TSPT).

Inbred lines	Sub-Group	Infected incidence (%)	PZA00672.8	PZE-105072717
**96201**	P	18.3	GG	AA
**Han21**	P	32.6	GG	AA
**K36**	P	42.0	GG	AA
**L005**	P	28.1	GG	AA
**L069**	P	22.9	GG	AA
**Nongda178**	P	64.8	GG	AA
**P138**	P	52.0	GG	AA
**Qi318**	P	6.1	GG	AA
**Qi319**	P	8.0	GG	AA
**Shen135**	P	10.2	GG	AA
**Shen137**	P	6.1	GG	AA
**Song1145**	P	90.3	GG	AA
**Wu125**	P	11.7	GG	AA
**X3514**	P	54.0	GG	AA
**Zong548.1521**	P	12.0	GG	AA
**Zun90110**	P	28.5	GG	AA
**72–125**	TSPT	54.2	AA	GG
**Chang7.2**	TSPT	80.8	AA	GG
**HR962**	TSPT	66.2	AA	GG
**Huangyesi3**	TSPT	72.6	AA	GG
**Huangzaosi**	TSPT	64.4	AA	GG
**Luyuan133**	TSPT	58.3	AA	GG
**X84.126.15.1**	TSPT	28.8	AA	GG
**X897**	TSPT	77.4	AA	GG
**Zhonger_O2**	TSPT	59.5	AA	GG
**444**	TSPT	47.1	GG	GG
**H21**	TSPT	70.4	GG	GG
**S001**	TSPT	65.7	GG	GG
**Bai197**	TSPT	73.3	AA	AA
**K12**	TSPT	72.2	AA	AA
**Tangsipingtou**	TSPT	42.7	AA	AA
**Wutang448**	TSPT	56.0	AA	AA
**Ji833**	TSPT	31.5	GG	AA
**X75.14Gao**	TSPT	22.0	GG	AA
**Yan38**	TSPT	43.1	GG	AA

## Discussion

### Genetic maps constructed from the GBS sequencing data greatly improves the mapping resolution of head smut resistance QTL

High density markers can greatly facilitate the identification of recombinant events and the exact recombinant breakpoints, which significantly improves the resolution of QTL mapping. In rice, using the genetic maps from genome sequencing, two major QTL for grain length and width could be directly mapped to the intervals of less than 200 kb region containing their responsible genes [37–38]. In maize, using the genetic maps from the high density GBS markers, the silk color regulating gene *r1* was mapped to a 700 kb interval [39].

In the current study, the genetic maps of bin markers for all 10 chromosomes were constructed with GBS data. By using the constructed genetic map, the major head smut resistant QTL *q2*.*09HR*, designated as *qHSR1* or *qHS2*.*09* in previous studies [12–14], was mapped to the marker region of a 1.4 Mb using a RIL population with 143 lines. This QTL largely overlaps with the fine mapped resistant *qHS2*.*09* by Weng et al. [14]. Therefore, the genetic map, constructed from low-coverage GBS data, has shown great promise in improving the mapping resolution of QTL.

### Head smut resistance by QTL *q5*.*03HR* is consistent

Thus far, more than twenty head smut resistant QTL have been identified across all 10 maize chromosomes [2, 10–14]. In our study, one head smut resistance QTL *q5*.*03HR* was mapped to the region of 70.8–80.8 Mb on Chromosome 5 with a RIL population. Compared with previous reports, only one head smut resistance QTL had been mapped to Chromosome 5 with very low resolution [10]. Either *q5*.*03HR* represents the confirmation of the previously reported Chromosome 5 QTL but with greatly improved mapping resolution, or it is a novel QTL for head smut resistance.

MAS and backcross populations have been widely used in QTL validation, fine mapping and gene cloning in maize and other crops [14, 40–43]. In such a population, the target QTL becomes the major source of genetic variation along with the gradual elimination of the background “noise” by continuous backcross and MAS [43]. In this study, a series of backcross populations were developed for QTL validation. The results showed that the head smut resistance QTL *q5*.*03HR* could steadily be detected among all the BC populations. By integrating the mapping results of the RIL and backcross populations, the interval of *q5*.*03HR* could be narrowed down to a region of 70.8–78.4 Mb. The identification of more recombination events within this region will be one of the determining factors for the fine mapping or cloning of *q5*.*03HR*.

### Head smut resistance QTL were validated and further delimited by association analysis

The approach of association mapping provides great opportunities to use historical recombination events for the genetic dissection of complex traits, especially for those species with rapid LD decay [44–46]. In our study, within the region of *q2*.*09HR*, four SNPs are significantly associated with head smut resistance (Table 3, Fig 5C). It was also observed that these four SNPs are located in a 1Mb interval, which is within a previously described interval and two of them exactly overlap with the previous study (PZA00672.8 and PZE-102188421) [14]. Thus, by the combined strategy of linkage mapping and association analysis, the major head smut resistant QTL *q2*.*09HR* or *qHSR1* was defined to an interval of sub Mb region.

It has been well known that the Lancaster-like inbred lines derived from Mo17 generally possess high level of head smut resistance [12]. In fact, the Mo17 haplotype in the region of *q2*.*09HR* is the main source of head smut resistance for these Lancaster inbred lines [14]. In our study, the favorable haplotype of *q2*.*09HR* from the resistant parent “QI319” is different from that of Mo17 based on genotypic data from MaizeSNP50 BeadChip. As such, there seems to be more than one head smut resistance haplotype at *q2*.*09HR*.

By the QTL mapping with a RIL population, *q5*.*03HR* was mapped to a marker interval of about 10 Mb on Chromosome 5. Only limited improvement in mapping resolution was achieved with a series of backcross populations. *q5*.*03HR* is located very close to the centromere, a region with low recombinantion frequency. Identifying more recombinants becomes one of the key limitations for the fine mapping of *q5*.*03HR*. Association analysis, which takes advantage of historical recombination events, would not only validate the QTL, but also improve its mapping resolution [44–46]. In this study, one associated SNP (PZE-105072717) within the *q5*.*03HR* region was detected. Meanwhile, LD analysis within *q5*.*03HR* revealed that the most significant SNP (PZE-105072717) was located with a 970 kb LD block (Fig 6). When conducting GWAS, the disequilibrium between multiple factors affecting a trait might bring the false association [47]. As such, the results of independent studies were expected to provide validation opportunities. Compared with the previous studies, weakly associated signals for the region of *q5*.*03HR* had also been observed although none of them beyond the threshold [14, 17]. Therefore, combining the linkage results, we suppose that the regulator underlying *q5*.*03HR* might be located around the LD region covering the most associated SNP (PZE-105072717) on Chromosome 5.

### Both the additive and epistastic effects determine the regulation of head smut resistance in maize

In the present study, we have shown that the pyramiding of beneficial alleles for both QTL enhances head smut resistance (Fig 3, Table 4). No epistasis could be detected among those two resistant loci. According to the simple additive model, all the detected associated SNPs only explained 43.1% of the total phenotypic variation, which was much lower than the value of broad-sense heritability (88.7%). Meanwhile, a total of 58 significant interaction pairs between the associated SNPs were detected. When the genetic effects of this part were considered, the full model could explain 64.1% of the total phenotypic variation. Thus, epistastic effects among the head smut resistance-related loci also played the important role in the regulation of head smut resistance in maize. Moreover, some of the detected SNPs showed complex epistastic interactions with other loci, such as the most significantly SNP (PZA00672.8) within *q2*.*09HR* significantly interacted with other 10 associated SNPs across the genome. The other two SNPs (PZE-104087413 on Chromosome 4 and PZE-108094946 on Chromosome 8) significantly interacted with nine other associated SNPs. Different from other studies, such as the genetic architecture of southern leaf blight and southern leaf blight in nested association mapping population [18–19], we postulated that both the additive and epistastic effects determine the regulation of head smut resistance in maize. Moreover, because quantitative resistance is usually more durable than qualitative resistance [48], more genes/QTL should be detected, confirmed and applied for the future assisted selection in maize breeding.

## Supporting Information

S1 File Inbred list of association panel with the information of population structure and the BULPs of head smut infected incidence across two environments. (XLS)

S2 File Significantly associated SNPs detected by GWAS of association panel with 277 inbred lines. (declared by the threshold of *P* < 3.61×10^−6^)
(XLS)

S3 File Significantly interacted pairs among the most associated SNPs within 1 Mb window size. (XLS)
